# The Protective Effect of* Sonneratia apetala* Fruit Extract on Acetaminophen-Induced Liver Injury in Mice

**DOI:** 10.1155/2019/6919834

**Published:** 2019-06-19

**Authors:** Jingjing Liu, Dandan Luo, Yulin Wu, Changjun Gao, Guosheng Lin, Jinfen Chen, Xiaoli Wu, Qian Zhang, Jian Cai, Ziren Su

**Affiliations:** ^1^Mathematical Engineering Academy of Chinese Medicine, Guangdong Provincial Key Laboratory of New Drug Development and Research of Chinese Medicine, Guangzhou University of Chinese Medicine, Guangzhou 510006, China; ^2^Guangdong Provincial Key Laboratory of Silviculture, Protection and Utilization, Guangzhou 510520, China; ^3^Postdoctoral Programme, The Second Affiliated Hospital of Guangzhou University of Chinese Medicine, Guangzhou 510006, China; ^4^Guangdong Academy of Forestry, Guangzhou 510520, China

## Abstract

Acute liver injury is a common consequence of taking overdose of acetaminophen (APAP). The aim of this study was to evaluate the antioxidant activity and hepatoprotective effect of a mangrove plant* Sonneratia apetala* fruit extract (SAFE) on APAP-induced liver injury in mice. Mice were orally pretreated with SAFE (100, 200, and 400 mg/kg) daily for one week. The control and APAP groups were intragastrically administered with distilled water, and NAC group was treated with N-Acetyl-L-cysteine (NAC) before APAP exposure. The results manifested that SAFE significantly improved survival rates, attenuated hepatic histological damage, and decreased the alanine aminotransferase (ALT) and aspartate aminotransferase (AST) levels in serum in APAP-exposed mice. SAFE treatment also increased glutathione (GSH) level and glutathione peroxidase (GSH-Px) activity, enhanced catalase (CAT), and total antioxidant capacity (T-AOC), as well as reducing malondialdehyde (MDA) level in liver. In addition, the formation of tumor necrosis factor-alpha (TNF-*α*), interleukin 6 (IL-6), and elevation of myeloperoxidase (MPO) in APAP-exposed mice were inhibited after SAFE treatment. And SAFE also displayed high DPPH radical scavenging activity and reducing power* in vitro*. The main bioactive components of SAFE such as total phenol, flavonoid, condensed tannin, and carbohydrate were determined. The current study proved that SAFE exerted potential protective effect against APAP-induced acute liver injury, which might be associated with the antioxidant and anti-inflammatory activities of SAFE.

## 1. Introduction

Acetaminophen (N-acetyl-para-aminophenol, APAP), a classical antipyretic and analgesic, is widely used to treat cold and fever clinically all over the world, but overdose of APAP engenders acute liver injury and even more severe liver failure [1]. According to a report, APAP-induced hepatotoxicity is the primary cause of acute liver injury and results in about 50% of all the cases in developed countries [2]. The basic mechanism of acute liver injury induced by APAP consists of reactive metabolites production, GSH depletion, and protein adducts generation that further contribute to the dysfunction of mitochondria, the production of peroxynitrite, nuclear DNA fragmentation and ultimately lead to hepatocellular necrosis [3, 4]. The clinical antidote to APAP toxicity is N-Acetyl-L-cysteine (NAC), which is an available nonspecific antioxidant that alleviates oxidative stress in the manner of direct obliteration or serves as a glutathione precursor to afford sufficient glutathione to combine with N-acetyl-p-benzoquinone imine (NAPQI) [5, 6]. NAC is considered as a safe and effective antidote for hepatotoxicity caused by APAP overdose and can be optimally used within 8 h [7]. However, NAC also has some side effects, including headache, nausea, vomiting, diarrhea, and flatulence [6]. Hence, it is still a focused issue to be solved that to develop safe and effective drugs against acute liver injury caused by APAP.

In recent years, the use of plants in treating diseases has attracted more attentions due to economic price as well as little side effects [8, 9].* Sonneratia apetala *(*S. apetala*), also called Keora in local, is a member of the Lythraceae belonging to mangrove, which widely exists in Sundarbans (the world's largest mangrove forest) and the coastal regions in Bangladesh, India, Myanmar, Malaysia, New Guinea, China,* etc. *[10–12]. As an exotic mangrove species introduced from India, Bengal, and Sri Lanka [13],* S. apetala *mainly distributes in the southeast coastal areas in China [14] and serves as the dominant species of mangroves because of its high adaptability, rapid growth, high seed setting rate, and other characteristics [15]. The fruit of* S. apetala *is edible and generally made into food with multifarious preparing processes by people in coastal Bangladesh, India, Myanmar, and China, which are also processed into market products such as sour sauce, fermented juice, soft drinks, and so on [16, 17]. Moreover, it also exerts pharmaceutical value and is often used as medicine to treat illnesses including hepatitis, dysentery, bruise,* etc.* by people in local [16–19]. The fruit of* S. apetala* is applied to curing sprain and cough in China, and it is also used as medicine for internal diseases along with leaf and flower [20]. It has been proved that the fruits and barks of* S. apetala* display the efficacy of curing wheezing, fevers, ulcers, swellings, sprains as well as hemorrhage [21]. Moreover,* S. apetala* is used as anti-inflammatory agents to treat gastrointestinal disorders such as dysentery, diarrhea, and stomachache by folk medicinal healers in Bangladesh [16, 17]. Extracts of* S. apetala* fruits have showed a wide range of biological activities, including antioxidant, antidiabetic, anticancer, and antibacterial activities [10, 22]. The main bioactive constituents of* S. apetala *are polyphenols, flavonoids, tannins, and carbohydrates, which contribute to its antioxidant activity and make it to be a potential source of natural antioxidants [11, 23–26]. Additionally, triterpenes, steroids, carboxylic acids, and lactones also contribute to* S. apetala* biological properties [10, 18, 26]. Liver damage in most situations involves oxidative stress that can directly participate in liver pathologies and their processes [27]. Overdose APAP overwhelms the hepatic detoxification process and depletes GSH, which leads to oxidative stress and further contributes to liver injury [28]. Therefore, antioxidants and antioxidant-rich plant may be used as therapeutic agents for liver damage [27, 29, 30]. An increasing body of evidences have proved that alleviating oxidative stress and inflammation would be a potential way to attenuate acute liver injury induced by APAP [31–33]. Hence, as a potential natural antioxidant,* S. apetala *may play an important role in treating APAP-induced acute liver injury. Although the antioxidant and anti-inflammatory activities of* S. apetala* have been reported, its hepatoprotective activity has rarely been investigated. Therefore, the aim of the present study was to assess the antioxidant activity and hepatoprotective effect of* S. apetala* fruit extract (SAFE) against APAP-induced acute liver injury in mice.

## 2. Materials and Methods

### 2.1. Materials

The fresh* S. apetala *fruits of the mangrove plant were collected from Nansha Coast Wetland Scenic spot, Guangzhou, China. APAP (CAS: 103-90-2), NAC (CAS: 616-91-1), and ascorbic acid (CAS:50-81-7) were obtained from Guangzhou Feibo Biological Technology Co., Ltd. 1,1-Diphenyl-2-picryl-hydrazyl (DPPH, CAS:1898-66-4), (+)-Catechin (CAS:154-23-4), and Folin-Ciocalteu reagent (CAS:NONE6060) were purchased from Shanghai Macklin Biochemical Co., Ltd. Potassium ferricyanide (CAS:13746-66-2), trichloroacetic acid (CAS:76-03-9), ferric trichloride (CAS:10025-77-1), and vanillin (CAS:121-33-5) were obtained from Damao Chemical Reagent Factory China. Gallic acid was purchased from Wenzhou Ouhai Fine Chemical Co., Ltd. (Zhejiang, China). The alanine aminotransferase (ALT) detection kit (C009-2), aspartate aminotransferase (AST) detection kit (C010-2), glutathione (GSH) assay kit (A006-2), glutathione peroxidase (GSH-Px) assay kit (A005), catalase (CAT) assay kit (A007-1), total antioxidant capacity (T-AOC) assay kit (A015), malondialdehyde (MDA) assay kit (A003-1), and myeloperoxidase (MPO) detection kit (A044) were purchased from Nanjing Jiancheng Bioengineering Institute (Nanjing, China). The enzyme linked immunosorbent assay (ELISA) kits for determination of inflammatory cytokines TNF-*α* (E-20219) and IL-6 (E-20012) were obtained from Beijing Andy Huatai Biological Technology Co., Ltd. The other analytical reagents and chemicals were obtained from Tianjin Zhiyuan Chemical Reagent Co., Ltd.

### 2.2. Extraction of the Fruits of S. apetala

The fresh* S. apetala* fruits (5 kg) were collected and rinsed thoroughly before extraction and then extracted with 2-fold boiled water for 3 h; occasional stirring was performed to prevent from burning. Extract was obtained and concentrated to 1.1 L after filtration in vacuum. Then, the concentrated sample was freeze-dried. The SAFE was obtained and stored at 4°C for further analysis.

### 2.3. Chemical Compositions Analysis

#### 2.3.1. Phytochemical Analysis

The qualitative phytochemical assays were carried out to evaluate the presence of flavonoids, phenols, tannins, saponins, cardiac glycosides, steroids, alkaloids, anthraquinones, organic acids, carbohydrates, protein, and amino acids in SAFE using standard procedures [10, 34].

#### 2.3.2. Determination of Total Phenol Content

The total phenol content of SAFE was determined by Folin-Ciocalteu reagent [35] using gallic acid as the standard. Briefly, 150 *μ*L samples in different concentrations were mixed with 750 *μ*L of Folin-Ciocalteu reagent and 600 *μ*L of sodium carbonate (7.5%,* w/v*). Then the mixture was incubated at 40°C for 30 min and we measured the absorption at 760 nm.

#### 2.3.3. Determination of Total Flavonoid Content

The measurement of total flavonoid content was performed by the aluminium chloride colorimetric method [36, 37]. In brief, the total volume of reaction solution was 2 mL including 200 *μ*L sample or standard ((+)-catechin), 60 *μ*L of 5% NaNO_2_ and 10% AlCl_3_, 400 *μ*L of NaOH (1M), and 480 *μ*L distilled water. The absorbance measurements were performed at 510 nm.

#### 2.3.4. Determination of Total Condensed Tannins Content

The detection of total tannins content in SAFE was carried out according to the reported method [30] using (+)-catechin as a standard. Briefly, 0.5 mL sample or standard was mixed with 3 mL of vanillin (4%,* w/v*) in methanol and 1.5 mL concentrated HCl following stand at room temperature for 15 min. Then the absorbance at 500 nm was determined against methanol as a blank.

#### 2.3.5. Determination of Total Carbohydrate Content

The total carbohydrate content of SAFE was determined by phenol-sulfuric acid method according to reported method [38, 39] using glucose as the standard. In brief, 2 mL of sample solution was mixed with 1 mL of phenol (5.0%,* w/v*) and 5 mL of concentrated sulfuric acid. Then the absorbance of mixture was recorded at 490 nm after standing at room temperature for 15 min.

### 2.4. Assay of Antioxidant Activity of SAFE In Vitro

#### 2.4.1. DPPH Radical Scavenging Activity

The scavenging activity of DPPH radical was determined by reported method [40]. Briefly, 1 mL of sample solution with different concentrations (20, 40, 60, 80, 100, and 120 *μ*g/mL) was added to 2 mL of freshly prepared DPPH, mixing thoroughly, and the mixture was incubated at room temperature for 30 min in the dark. The absorbance of mixture was recorded at 517 nm. The DPPH radical scavenging activity was calculated with the following equation:

(1) DPPH  radical  scavenging  activity  %=1−A0 -A1A2×100%

where A_0_ was the absorbance of a mixture of sample and DPPH solution, A_1_ was the absorbance of sample solution with ethanol, and A_2_ was the absorbance of a mixture of DPPH solution and distilled water. The ascorbic acid (Vc) was served as positive control.

#### 2.4.2. Reducing Power

The reducing power of SAFE was measured according to the reported method [41]. 2.5 mL of phosphate buffer (0.2 M, pH 6.6) and 2.5 mL of potassium ferricyanide (1.0%,* w/v*) were mixed with different concentrations SAFE solution and incubated at 50°C for 20 min. Then 2.5 mL trichloroacetic acid (10%,* w/v*) was added to the mixture prior to centrifugation at 1500 g for 10 min. The 2.5 mL of supernatant was mixed with 0.5 mL of ferric trichloride (1.0%,* w/v*) and 2.5 mL of distilled water, and the absorbance was detected at 700 nm. Vc was used as positive control.

### 2.5. Assay of Hepatoprotective Effect of SAFE In Vivo

#### 2.5.1. Animals

Male Kunming mice (8 weeks old) were purchased from Guangzhou University of Chinese Medicine. These mice were domesticated under the environment with free access to food and water (temperature: 18~29°C, humidity: 40%~70%, 12 h light and dark cycle) for 1 week before experiment. All experimental procedures were performed according to the Ethics Committee for the Welfare of Experimental Animals of Guangzhou University of Chinese Medicine (no. 2016047).

#### 2.5.2. Experimental Design

The APAP-induced liver injury in mice was established by injecting 220mg/kg of APAP according to the publications [2, 42]. All the mice were randomly assigned to the following six groups (n = 15/group): (1) control group, (2) APAP group (APAP, 220 mg/kg), (3) NAC group (220 mg/kg APAP + 200 mg/kg NAC), (4) low dose of SAFE group (220 mg/kg APAP + 100 mg/kg SAFE), (5) middle dose of SAFE group (220 mg/kg APAP + 200 mg/kg SAFE), and (6) high dose of SAFE group (220 mg/kg APAP + 400 mg/kg SAFE). Different doses of SAFE were dissolved in suitable amount of distilled water. APAP was dissolved in normal saline solution before being intraperitoneally administered to mice. And the NAC, serving as positive control, accepted equal treatment with SAFE. For groups 1 and 2, distilled water was administered orally every day for 7 days. At the same time, group 3 were treated with NAC. For group 4-6, mice were intragastrically given 100, 200, and 400 mg/kg of SAFE, respectively. 4 h after the last administration, all the mice were given APAP by intraperitoneal injection, while the control group was injected with the same volume of normal saline. 12 h after the APAP challenge, blood samples were collected from the mice eye socket vein and centrifuged (3000 rpm, 4°C, 10 min) to obtain serum. Then mice were sacrificed and liver tissues were harvested, washed with normal saline three times, and stored at -80°C for further analysis.

#### 2.5.3. Survival Analysis

In order to estimate mortality in APAP-exposed mice, another 90 mice were treated as above. All the groups were fasted for 12 h and subsequently fed normal diet. The survival situations of mice were recorded every 12 h for 5 days to obtain survival rates.

#### 2.5.4. Histopathology

The collected liver tissues from all the groups were fixed in 4% paraformaldehyde, dehydrated and embedded in paraffin, sectioned at 5 *μ*m thickness, and dyed with hematoxylin and eosin (H&E) routinely. Liver histopathologic changes were captured with an optical microscope at 200× magnification (E100, Nikon Corporation).

#### 2.5.5. ALT and AST Levels Assay

The blood samples were kept at room temperature for 2 h and then centrifuged at 3,000 rpm for 10 min at 4°C to obtain serum. ALT and AST levels in the serum were quantified using commercial kits.

#### 2.5.6. GSH, GSH-Px, CAT, T-AOC, and MDA Assay

The liver tissues were thawed and homogenized in 9-fold (g: mL) of ice-cold normal saline. The homogenate was centrifuged at 3000 rpm for 10 min at 4°C to obtain supernatant. The levels of GSH, GSH-Px, MDA, CAT, and T-AOC in supernatant were measured using commercial kits according to the manufacturer's protocols.

#### 2.5.7. MPO Activity Assay

The liver samples were weighted accurately and homogenized with precold homogenization medium (1:19). Then, the MPO activity in liver tissue was measured using commercial kit according to the manufacturer's method.

#### 2.5.8. TNF-*α* and IL-6 Levels Assay

Liver tissues were weighted and homogenized with 9-fold ice-cold PBS (pH 7.4) at 2-8°C. Thereafter, liver tissue homogenates were centrifuged at the speed of 3000 rpm for 20 min. The assay of TNF-*α* and IL-6 was performed as followings. Hepatic homogenate samples and standards were added to the ELISA plates followed by incubation for 30 min at 37°C. Following washing for 5 times by wash solution, every well was incubated with 50 *μ*L of HRP-conjugate reagent at 37°C for 30 min followed by washing for 5 time as well. Subsequently, 50 *μ*L of chromogen solution A and B was, respectively, added to each well followed by incubation for 15 min at 37°C. Finally, 50 *μ*L of stop solution was added to the wells and the absorbance was read at 450 nm.

#### 2.5.9. Statistical Analysis

All data were represented as mean ± SD. The statistical analysis was performed by Statistical Product and Service Solutions software 23.0 (SPSS Inc., USA). One-way analysis of variance (ANOVA) followed by the multiple comparisons of Least Significant Difference (LSD) test was used for all the data analysis. The survival analysis was determined by Kaplan-Meier curve and log-rank Mantel-Cox test. In this study,* p* < 0.05 was considered as a significant difference.

## 3. Results

### 3.1. The Phytochemical Composition Analysis of SAFE

In this study, the dried extraction was obtained from the fruits of* S. apetala*. The yield of SAFE was approximately 1.28% (*w/w*).The phytochemical screening and chemical composition of SAFE are shown in Tables 1 and 2, respectively. The results showed the presence of flavonoids, phenols, tannins, steroids, alkaloids, organic acids, carbohydrates, protein, and amino acids on SAFE. The total phenol, flavonoid, and condensed tannin content on SAFE were expressed in mg gallic acid equivalent per g of extract and mg (+)-catechin equivalent per g of extract, respectively. SAFE exhibited the total phenol content of 143.64 ± 6.49 mg/g, the total flavonoid content of 20.93 ± 0.79 mg/g, and the total condensed tannin content of 5.47 ± 0.25 mg/g. It was also found that the total carbohydrate content of SAFE was 23.94 ± 0.50%.

### 3.2. Antioxidant Activity In Vitro

**Figure 1:**
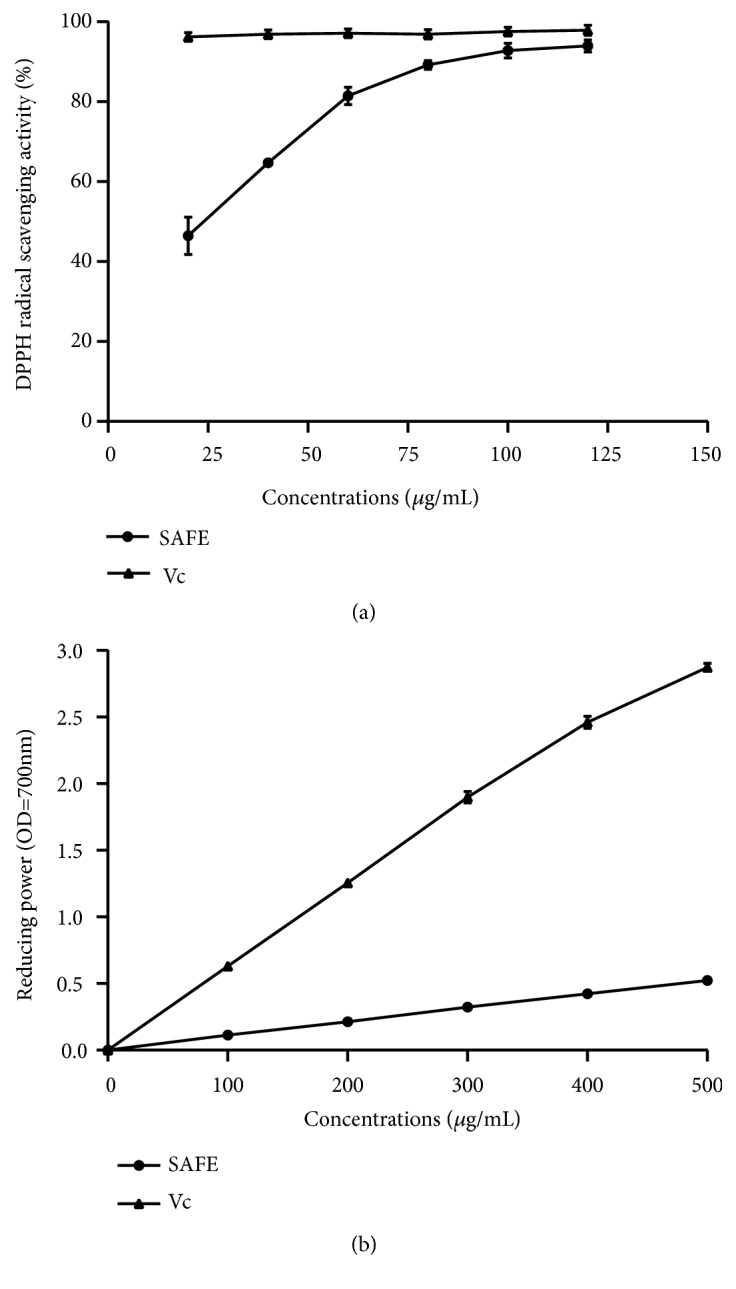
Antioxidant activity of SAFE* in vitro*. (a) DPPH radical scavenging activity and (b) reducing power. Data are presented as a mean ± SD (n = 3).

The DPPH radical scavenging activity of SAFE is shown in Figure 1(a). SAFE revealed strong scavenging activity on DPPH with the maximum suppression of 93.91% at the concentration of 120 *μ*g/mL, and the IC_50_ was measured to be 23.65 *μ*g/mL. As shown in Figure 1(b), the reducing power of the tested samples steadily raised with the increasing sample concentration. At 500 *μ*g/mL, the reducing power was 0.52 for SAFE.

### 3.3. The Hepatoprotective Effect of SAFE In Vivo

#### 3.3.1. SAFE Attenuated APAP-Induced High Mortality Rate

**Figure 2:**
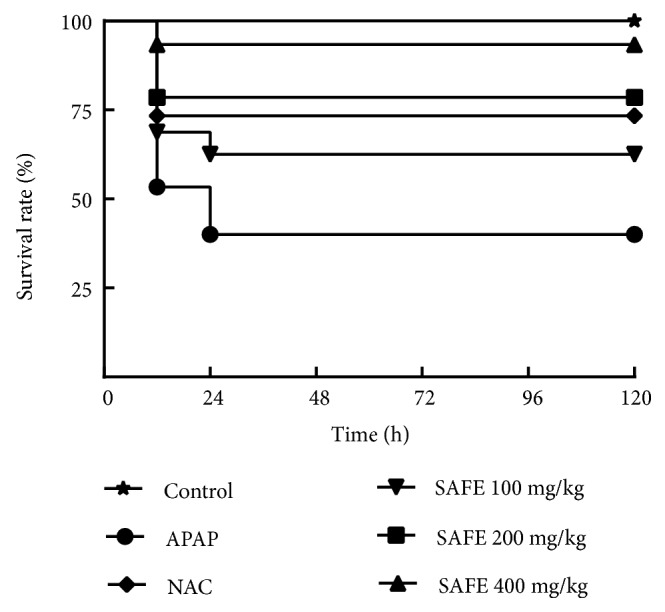
SAFE attenuated APAP-induced high mortality rate. The survival rate was expressed as Kaplan-Meier curves followed by the log-rank Mantel-Cox test for comparison among curves (n =15). Survival rate was significantly lower in APAP group mice compared to that in control, 200 mg/kg SAFE and 400 mg/kg SAFE groups (*p* < 0.01,* p* < 0.05 and* p* < 0.01, respectively).

As displayed in Figure 2, the survival rate at 12 h was about 53.33% and finally dropped to only 40% at 120 h after APAP exposure in APAP group. Interestingly, the survival rate of NAC-treated mice was 73.33% at 12 h and maintained the same until 120 h. And SAFE treatment (100, 200, and 400 mg/kg) significantly increased the survival rates (60%, 80%, and 93.33%) at 120 h compared with APAP group (*p *< 0.05).

#### 3.3.2. SAFE Relieved Histopathological Damage Induced by APAP

**Figure 3:**
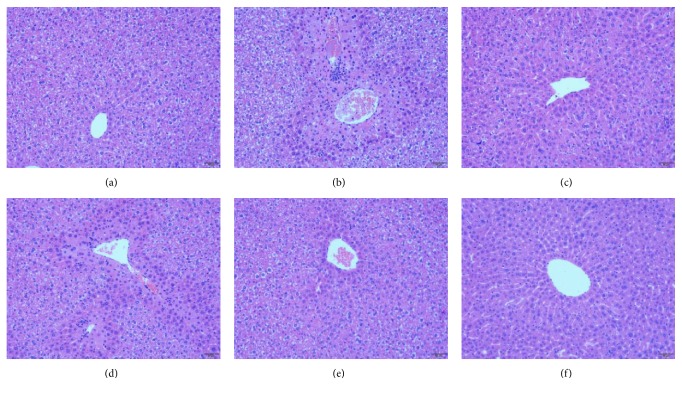
SAFE relieved hepatic histopathological damage in APAP-treated mice. (a) Control; (b) APAP; (c) NAC; (d) 100 mg/kg of SAFE; (e) 200 mg/kg of SAFE; (f) 400 mg/kg of SAFE. Scale bar: 50 *μ*m.

As shown in Figure 3, hepatic cells of control group expressed normal histology. However, hepatic sections of APAP-treated mice showed hepatocyte necrosis, inflammation cell infiltration, and congestion in central vein while compared with control group. The histopathological damage was alleviated with SAFE and NAC treatment.

#### 3.3.3. SAFE Mitigated APAP-Induced Hepatotoxicity

**Figure 4:**
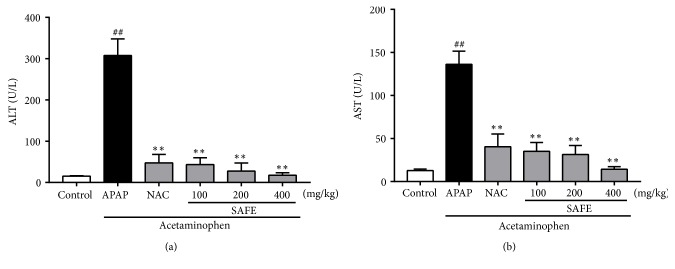
SAFE inhibited ALT (a) and AST (b) levels in serum. Data are presented as a mean ± SD (n = 8), ^##^*p* < 0.01 vs. control group; *∗p* < 0.05 or *∗∗p* < 0.01 vs. APAP group.

The protective effect of SAFE on APAP-induced liver injury was evaluated by measuring the ALT and AST levels in serum. The ALT (Figure 4(a)) and AST (Figure 4(b)) levels in APAP group were 307.67 ± 40.10 U/L and 151.53 ± 8.92 U/L after APAP exposure, respectively, which were 19.93-fold and 11.96-fold higher than that of the control groups, respectively. However, SAFE treatment dose-dependently inhibited the ALT and AST levels. Compared with APAP group, ALT levels in 100, 200, and 400 mg/kg groups declined to 43.16 ± 16.78 U/L, 27.42 ± 19.66 U/L, and 17.65 ± 3.95 U/L, respectively, and the AST levels declined to 111.73 ± 8.08 U/L, 50.08 ± 7.06 U/L, and 21.72 ± 1.97 U/L, respectively. Among them, 400 mg/kg of SAFE treatment displayed the best effects with the similar ALT and AST levels as that of control group.

#### 3.3.4. SAFE Restrained APAP-Induced Liver Oxidative Stress

**Figure 5:**
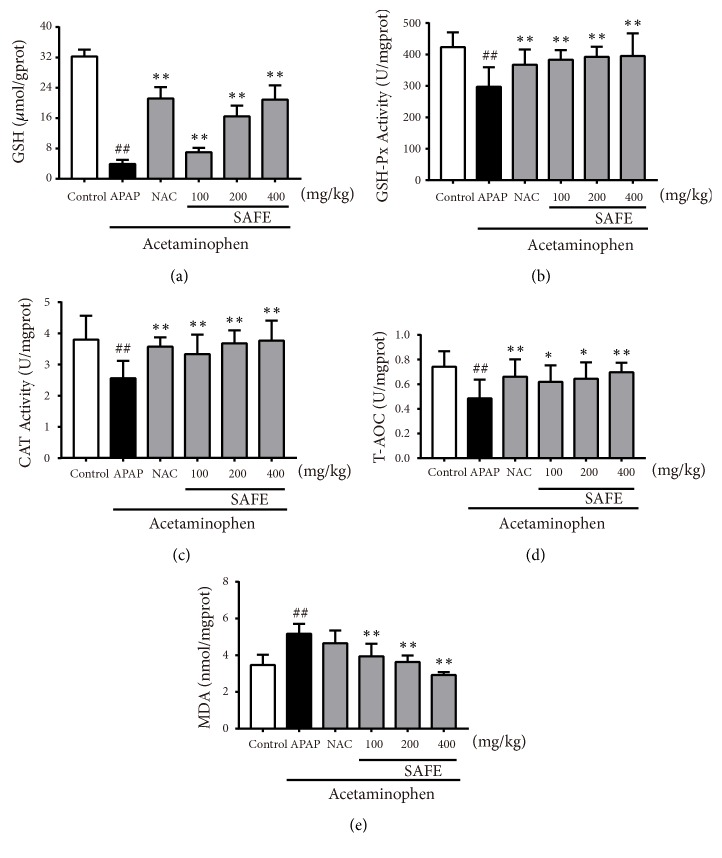
SAFE restrained APAP-induced liver oxidative stress in APAP-treated mice. (a) GSH; (b) GSH-Px; (c) CAT; (d) T-AOC; and (e) MDA. Data are presented as a mean ± SD (n = 8), ^##^*p* < 0.01 vs. control group; *∗p* < 0.05 or *∗∗p* < 0.01 vs. APAP group.

As shown in Figure 5, 12 h after APAP injection, GSH level and GSH-Px activity in liver decreased by 87.88% and 29.75% in APAP group when compared with control group (*p *< 0.01). However, SAFE treatment significantly attenuated APAP-induced GSH depletion and decreased GSH-Px activity (*p *< 0.01). Likewise, the CAT activity and T-AOC level also decreased by 32.63% and 35.14% in APAP-treated mice, and CAT activity increased by 30.47%, 43.36%, and 46.88% with SAFE (100, 200, and 400 mg/kg) treatment dose-dependently (*p *< 0.01), and T-AOC levels increased by 29.17%, 33.33%, and 43.75% with SAFE treatment (100, 200, and 400 mg/kg) as compared with APAP control (*p *< 0.05 or* p *< 0.01). Hepatic MDA levels also increased significantly by 48.99% in APAP-treated group but decreased significantly with SAFE treatment.

#### 3.3.5. SAFE Attenuated APAP-Induced Inflammation

**Figure 6:**
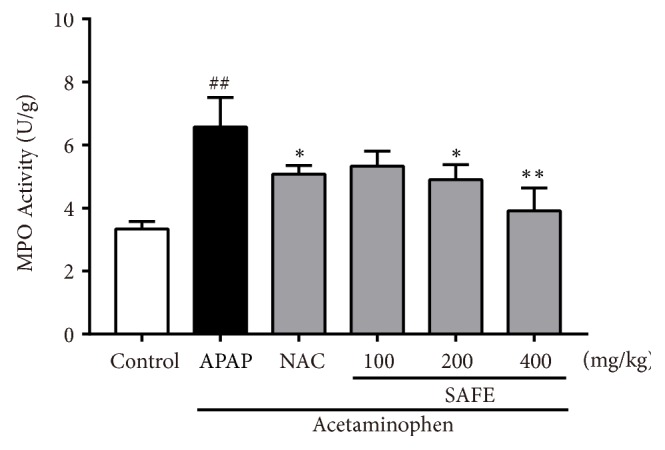
SAFE suppressed APAP-induced MPO regulation. Data are presented as a mean ± SD (n = 8), ^##^*p*< 0.01 vs. control group; *∗p* < 0.05 or *∗∗p* < 0.01 vs. APAP group.

**Figure 7:**
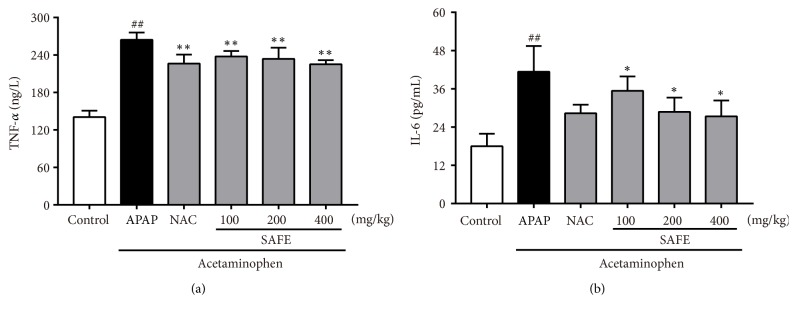
SAFE inhibited APAP-induced inflammation in mice. The level of TNF-*α* (a) and IL-6 (b) was measured. Data are presented as a mean ± SD (n = 8), ^##^*p* < 0.01 vs. control group; *∗p* < 0.05 or *∗∗p* < 0.01 vs. APAP group.

As shown in Figure 7, the hepatic TNF-*α* and IL-6 levels increased significantly after APAP exposure. However, SAFE treatment significantly decreased the over production of TNF-*α* and IL-6 (*p* < 0.05). Furthermore, the MPO activity was also tested and results were shown in Figure 6. A single dose of APAP significantly elevated MPO activity in liver when compared with the control group (6.57 ± 0.94 vs. 3.34 ± 0.24 U/g,* p *< 0.01). But, SAFE treatment (200 and 400 mg/kg) significantly decreased the MPO activities compared with APAP group, respectively (4.90 ± 0.48 vs. 6.57 ± 0.94 U/g,* p *< 0.05; 3.91 ± 0.72 vs. 6.57 ± 0.94 U/g,* p *< 0.01).

## 4. Discussion

Recently, natural products from plants have been manifested to play basic roles in human health care and liver diseases on account of their biological activities [43]. Among them, phytochemicals, the bioactive ingredients derived from plants, have beneficial effects on the prevention and treatment of diseases [11].* S. apetala* is a dominant exotic mangroves species introduced to China and its fruits have high nutritional value and exhibit various bioactivities [11]. In this study, the phytochemical constituents and the antioxidant activity as well as the hepatoprotective effect of* S. apetala* fruit extracts on liver injury caused by APAP in mice were evaluated.

It is well known that phenols, flavonoids, tannins, and carbohydrate are bioactive components of plants which show high antioxidant activity [11, 30, 44]. In this study, the results supported by Anha Afrin Shefa et al. [16] showed that different chemical compounds were present in SAFE, such as flavonoids, phenols, tannins, steroids, alkaloids, carbohydrates, and so on. The contents of total phenol and flavonoid from SAFE were lower than flower extract of* Millingtonia hortensis Linn*, a plant containing an abundant resource of flavonoids [45], while being higher than other mangrove plants [46, 47]. Moreover, SAFE showed lower condensed tannin content compared with true mangrove plants in South China [48]. And the carbohydrate content of SAFE was high in this study. This finding was similar with the published report [11].

Reactive oxygen species (ROS) are the inevitable products of oxidative metabolic processes, which lead to oxidative stress and cause liver damage that can be alleviated by antioxidants [49]. Hence, it is necessary to find safe and effective antioxidants. It has been reported that the capacity of natural products including phenols, flavonoids, polysaccharides,* etc.* in treating liver injury may be due to its antioxidant activity [44, 50–52]. In order to study the antioxidant activity of natural products, different assays based on chemical methods such as DPPH radical scavenging activity and reducing power have been applied [53, 54]. Owing to more stability, simplicity, and rapidity, DPPH assay was generally used to evaluate the free radical scavenging capacity of natural antioxidant [55]. In present study, SAFE exhibited scavenging activity on DPPH radical, which was in accordance with that reported by Hossain et al. [56]. Moreover, the reducing power was also determined to evaluate SAFE antioxidant capacity* in vitro*. The results showed that reducing property of SAFE increased in a dose manner. Although the incompetency of SAFE to compete with positive controls (ascorbic acid in DPPH radical scavenging ability and reducing power), it did possess antioxidant activity and may be regarded as potential natural antioxidant. The antioxidant capacities of SAFE might be attributed to the presence of phytochemicals such as phenols, flavonoids, tannins, and carbohydrates, which were in accordance with the researches of Raja et al. [30] and Hossain et al. [11].

Drug-induced liver injury is a main cause of liver diseases in clinic and the incidence of drug-induced liver injury seems to be on the rise along with the increase in the number of new drugs available [57]. Among the drug-induced liver injury, APAP-induced liver injury has taken the lead [58]. APAP is safe antipyretic analgesics in therapeutic doses but is a major exposed factor in acute liver injury once over dose [59]. An overdose APAP will bring about hepatocellular injury that leads to the elevation of serum aminotransferases level; hence serum levels of ALT and AST among liver enzymes are widely used as indicators of liver function [43, 60, 61]. In our study, the significantly increased serum aminotransferases levels proved the success of establishing APAP-induced liver injury model in mice. And SAFE pretreatment suppressed serum levels of ALT and AST, which also manifested its hepatoprotective capacity. The improvement of survival rates and attenuation of liver histological damage in APAP-induced liver injury model further validated that SAFE could alleviate liver injury. These results were confirmed by other previous findings, the increase of survival rate, decrease of serum markers, and alleviation of liver histological damage in APAP-induced liver damage after the treatment of different plant extracts [62, 63]. Normally, clinical dose of APAP mainly binds to glucuronic acid or sulfate in the liver and then is excreted into plasma or bile [64, 65]. However, a small proportion of APAP administered at clinical dose can be rapidly metabolized by cytochrome P450 (CYP) system in liver and subsequently forms available reactive metabolite, N-acetyl-p-benzoquinone imine (NAPQI), combined with GSH and detoxified to innoxious mercaptoacetic acid in physiological conditions [66]. Nevertheless, a noxious dose of APAP produces excessive NAPQI that consumes GSH by reacting with it and binding cysteine groups on hepatocytes, which results in the formation of protein adducts by binding redundant NAPQI and proteins in a covalent binding manner [67]. The formation of protein adducts means excess generation of reactive oxygen species and peroxynitrite beyond the antioxidant recovery system and finally leads to hepatocellular injury [68]. Wang et al. [69] proved that* Seabuckthorn Berry* polysaccharide pretreatment could alleviate APAP-induced oxidative stress by restoring GSH and increasing the activities of antioxidant enzymes. In this study, the hepatic GSH level and GSH-Px activity increased with SAFE treatment compared with APAP group. Moreover, SAFE treatment also increased CAT activity and T-AOC level, as well as decreasing MDA level. These results proved that SAFE attenuated APAP-induced liver injury by alleviating oxidant stress and improving antioxidant enzymes activity, which were also in accordance with other reports [33, 70, 71].

In addition, a quantity of inflammatory mediators such as cytokines and chemokines is also involved in the toxicity of APAP [67]. Several reports declared that APAP-induced liver injury might be attenuated by decreasing oxidative stress and inhibiting inflammatory response [72–74]. Alleviation of APAP-induced liver injury was able to be achieved by suppressing the inflammatory cytokines production, such as TNF-*α* and IL-6 [32, 75]. Salem et al. [33] demonstrated that* Phyllanthus muellarianus* aqueous leaf extract significantly reversed the increase of TNF-*α* and IL-6 in APAP-induced liver injury. In the present study, SAFE could alleviate inflammatory response in APAP-induced liver injury model via downregulating the levels of TNF-*α*, IL-6, and MPO activity, which might suggest that the anti-inflammatory effect was involved in the hepatoprotective effect of SAFE. Taken together, the study demonstrated that SAFE had the hepatoprotective effect against liver injury through alleviating oxidative stress and inflammatory response. However, it remains to be solved about the further mechanism of SAFE against APAP-induced liver injury.

## 5. Conclusion

In summary, the fruit extract obtained from* S. apetala* demonstrated the antioxidant activity, which might be related to its phenols, flavonoids, tannins, and carbohydrates contents. The pretreatment of SAFE exerted significant protective effects against APAP-induced acute liver injury and this may be associated with suppressing oxidative stress or inflammation. The findings of this present study support the fact that* S. apetala* is potential to be hepatic protective drug in the future. The actual bioactive components and underlying mechanisms of the hepatoprotective effect of* S. apetala* will be further in-depth study.

## Figures and Tables

**Table 1:** Phytochemical screening of fruit extract of *S. apetala*.

No.	Tests	Extract
1	Flavonoids	+
2	Phenols	+
3	Tannins	+
4	Saponins	-
5	Cardiac glycosides	-
6	Steroids	+
7	Alkaloids	+
8	Anthraquinones	-
9	Organic acids	+
10	Carbohydrates	+
11	Protein and amino acids	+

+: present; -: absent.

**Table 2:** The total phenols, flavonoids, condensed tannins, and carbohydrate content for SAFE.

Content	Total phenols (mg/g^ab^)	Total flavonoids (mg/g^ac^)	Total Condensed tannins (mg/g^ac^)	Total carbohydrates (*w*/*w*, %)

SAFE	143.64±6.49	20.93±0.79	5.47±0.25	23.94±0.50

^a^Average of 3 determinations, mean ± SD.  ^b^Gallic acid and  ^c^(+)-Catechin equivalent in mg/g of the extract.

## Data Availability

The data used to support the findings of this study are available from the corresponding author upon request.
